# Functional opsin patterning for *Drosophila* color vision is established through signaling pathways in adjacent object-detection neurons

**DOI:** 10.1242/dev.202388

**Published:** 2024-03-15

**Authors:** Manabu Kitamata, Yoshiaki Otake, Hideaki Kitagori, Xuanshuo Zhang, Yusuke Maki, Rika Boku, Masato Takeuchi, Hideki Nakagoshi

**Affiliations:** Graduate School of Environmental, Life, Natural Science and Technology, Okayama University, 3-1-1 Tsushima-naka, Kita-ku, Okayama 700-8530, Japan

**Keywords:** *Drosophila*, Eye, Opsin, Rhodopsin

## Abstract

Vision is mainly based on two different tasks, object detection and color discrimination, carried out by photoreceptor (PR) cells. The *Drosophila* compound eye consists of ∼800 ommatidia. Every ommatidium contains eight PR cells, six outer cells (R1-R6) and two inner cells (R7 and R8), by which object detection and color vision are achieved, respectively. Expression of opsin genes in R7 and R8 is highly coordinated through the instructive signal from R7 to R8, and two major ommatidial subtypes are distributed stochastically; pale type expresses Rh3/Rh5 and yellow type expresses Rh4/Rh6 in R7/R8. The homeodomain protein Defective proventriculus (Dve) is expressed in yellow-type R7 and in six outer PRs, and it is involved in Rh3 repression to specify the yellow-type R7. *dve* mutant eyes exhibited atypical coupling, Rh3/Rh6 and Rh4/Rh5, indicating that Dve activity is required for proper opsin coupling. Surprisingly, Dve activity in R1 is required for the instructive signal, whereas activity in R6 and R7 blocks the signal. Our results indicate that functional coupling of two different neurons is established through signaling pathways from adjacent neurons that are functionally different.

## Teaser

**Summary:** Dve activity is required for the proper opsin coupling of two neurons that is established through signaling pathways from adjacent neurons that are functionally different.

## INTRODUCTION

In vertebrates, rod cells express rhodopsin (Rh) and are involved in object detection in dim light, whereas cone cells, for example in humans, express one of three types of cone opsin, which absorb short (S, blue), medium (M, green), and long (L, red) wavelengths. Retinas have a mosaic distribution of these cone cells, and color vision is achieved by comparing the outputs of photoreceptor (PR) cells that have different spectral sensitivities (Nathans, 1999).

**Fig. 1.**
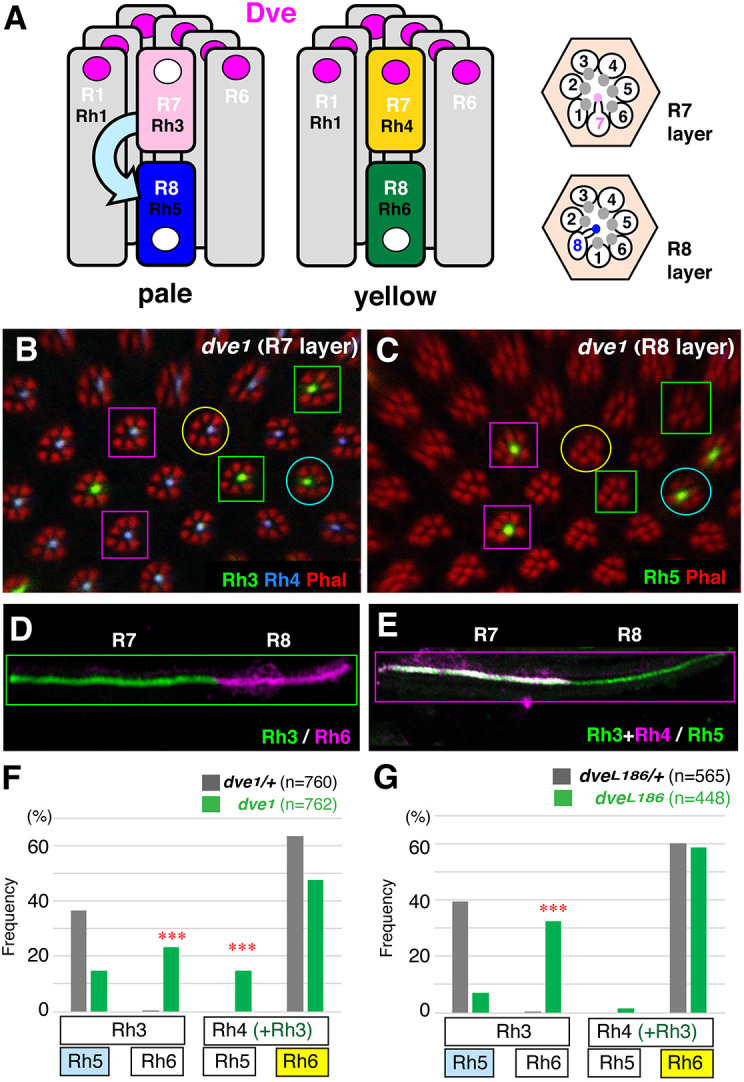
**Rhodopsin coupling is abnormal in *dve* mutant ommatidia.** (A) Schematic representation of rhabdomere arrangement of two major ommatidial subtypes (pale and yellow). Rectangles and circles represent rhabdomeres and nuclei of photoreceptor cells, respectively. Rhodopsin expression (Rh1, Rh3-Rh6) in rhabdomeres is shown in differently colored rectangles. Photoreceptor cells R1-R8 are shown in white text. Dve expression in nuclei is shown with magenta circles. The instructive Rh5-inducing signal from R7 to R8 is shown by a curved arrow. Horizontal sections of R7 and R8 layers (right) show the position of rhabdomeres and cell bodies. (B-F) Rhodopsin expression in *dve^1^* mutant eyes (y*w eyflp/Y; FRT42D dve^1^/FRT42D w*^+^*M*). (B,C) R7 and R8 layers of a *dve^1^* compound eye. Two typical types of ommatidia are outlined with circles [blue (pale) and yellow, Rh3/Rh5 and Rh4/Rh6, respectively]. Atypical types of ommatidia are outlined with squares (green and magenta, Rh3/Rh6 and Rh3+Rh4/Rh5, respectively). Rhabdomeres are labeled with Phalloidin (red). Expression of Rh3 and Rh5 (green), and Rh4 (blue) is shown. (D,E) Dissociated ommatidia show atypical Rhodopsin coupling as outlined with squares in B and C. Rh3 and Rh5 (green); Rh4 and Rh6 (magenta). (F) Rhodopsin coupling of *dve^1^/+* (*yw/Y; FRT42D dve^1^/+*, *N*=10 compound eyes, *n*=760 ommatidia) and *dve^1^/dve^1^* ommatidia (y*w eyflp/Y; FRT42D dve^1^/FRT42D w*^+^*M*, *N*=12, *n*=762). (G) Rhodopsin coupling of *dve^L186^/+* (*yw/Y; FRT42D dve^L186^/+, N*=7, *n*=565) and *dve^L186^/dve^L186^* ommatidia (y*w eyflp/Y; FRT42D dve^L186^/FRT42D w*^+^*M*, *N*=7, *n*=448)*.* Atypical couplings in *dve* mutant ommatidia are indicated by asterisks (***P<0.0001, chi-square test).

The *Drosophila* compound eye consists of ∼800 ommatidia. Every ommatidium contains eight PR cells: six outer cells (R1-R6) and two inner cells (R7 and R8). Outer PR cells express Rhodopsin1 (Rh1, also known as *ninaE*) and are involved in object (motion) detection, whereas color vision is achieved by inner PR cells, which express UV-sensitive opsins (Rh3 and Rh4) in R7, and blue- and green-sensitive opsins (Rh5 and Rh6) in R8 (Fig. 1A). Expression of opsin genes in R7 and R8 is highly coordinated through the instructive Rh5-inducing signal from R7 to R8 (Chou et al., 1999), and two major subtypes of ommatidium are distributed stochastically; pale type (∼30%) expresses Rh3/Rh5 and yellow type (∼70%) expresses Rh4/Rh6 in R7/R8 (Mollereau and Domingos, 2005; Wernet and Desplan, 2004). Photo-sensitive structure rhabdomeres of R7 and R8 are vertically aligned on the same axis in an ommatidium. Thus, this arrangement with functional rhodopsin coupling (Rh3/Rh5 and Rh4/Rh6) is thought to be crucial for color vision, namely the response to different wavelengths.

PR differentiation is regulated by two steps: (1) cell-fate determination and axonal projection during larval development, and (2) terminal differentiation such as rhabdomere morphogenesis and opsin gene expression during pupal development (Mollereau et al., 2001). The early event of terminal differentiation involves the specification of inner and R7 identities following the expression of *spalt* (*salm*) and *prospero* (*pros*) (Cook et al., 2003; Mollereau et al., 2001). Subsequent specification of R7 is caused by expression of *orthodenticle* (*otd*; *oc*) and *spineless* (*ss*) (Tahayato et al., 2003; Wernet et al., 2006). Expression of opsins in R7 and R8 is highly coordinated, and two major subtypes of ommatidium, pale and yellow, are established through the instructive signal from R7 to R8 within an ommatidium (Chou et al., 1999).

In response to R7 subtypes, transmission or blockade of the instructive signal is selected, and a bistable loop between *warts* (*wts*) and *melted* (*melt*) determines the state of Rh5 or Rh6 expression in R8 (Mikeladze-Dvali et al., 2005). Wts is a Ser/Thr kinase that is a core component of the Hippo signaling pathway involved in growth suppression. During ommatidial development, *wts* is necessary and sufficient for R8 to adopt the yellow-type identity, whereas *melt* plays the opposite role and induces the pale-type identity in R8. These two genes repress the transcription of each other to form a bistable loop (Anderson et al., 2017; Jukam and Desplan, 2011; Jukam et al., 2013; Thanawala et al., 2013). In addition, Activin and BMP signaling are required upstream of the Hippo pathway to establish expression of Rh5 or Rh6 in R8 (Wells et al., 2017). Involvement of Epidermal growth factor receptor, Rhomboid and Hibris is also reported (Birkholz et al., 2009a,b; Tan et al., 2020). However, mechanisms by which the instructive Rh5-inducing signal is transmitted from R7 to R8 remains unknown. Here, we provide evidence that transmission of the instructive signal is regulated by the activities of Defective proventriculus (Dve) in the outer PR cells, R1 and R6.

## RESULTS AND DISCUSSION

### Abnormal Rhodopsin coupling in *dve* mutant ommatidia

The homeodomain transcription factor Dve is involved in various functions including cell-type specification, functional differentiation and cell survival. Dve is expressed in all outer PR cells and in yellow-type R7 (yR7) (Fig. 1A). Dve expression in yR7 depends on the activity of Ss and represses the pale-type opsin Rh3 to specify the yellow-type identity. In *dve* mutant eyes, Rh3 and Rh4 are co-expressed in yR7 owing to derepression of Rh3 (Johnston et al., 2011). In *dve^1^* mutant eyes, the ratio of Rh5/Rh6 in the R8 layer was almost the same as that seen in controls; however, their coupling to R7 Rhodopsins was abnormal (Fig. 1B-F). Rh3/Rh6 coupling in R7/R8 is rarely observed in wild-type ommatidia, and this coupling is thought to be a default state. In *dve^1^* heterozygous control eyes, the Rh3/Rh6 coupling was observed only at 0.3%, whereas it was frequently observed at 23.1% in *dve^1^* homozygous mutant eyes (Fig. 1B-D,F). In addition, atypical coupling Rh3+Rh4/Rh5 was also observed at 14.8% (Fig. 1B,C,E,F). This atypical coupling is never observed in wild-type ommatidia. Thus, transmission of the instructive Rh5-inducing signal from R7 to R8 appears to be randomized in *dve^1^* mutant ommatidia. Because *dve^1^* is a severe loss-of-function allele, we checked the effect of null allele *dve^L186^*. Surprisingly, a considerable amount of *dve^L186^* mutant ommatidia (32.6%) exhibited atypical Rh3/Rh6 coupling, and almost all cells in R8 expressed Rh6 (Fig. 1G). In the absence of Dve activity, all R7 express Rh3 due to derepression of Rh3 in yR7. Thus, almost all ommatidia express Rh3 and Rh6 as a default state in *dve^L186^* mutant eyes, indicating that Dve activity is crucial for the instructive Rh5-inducing signal from R7 to R8.

### Dve activity in outer PRs regulates proper coupling

**Fig. 2.**
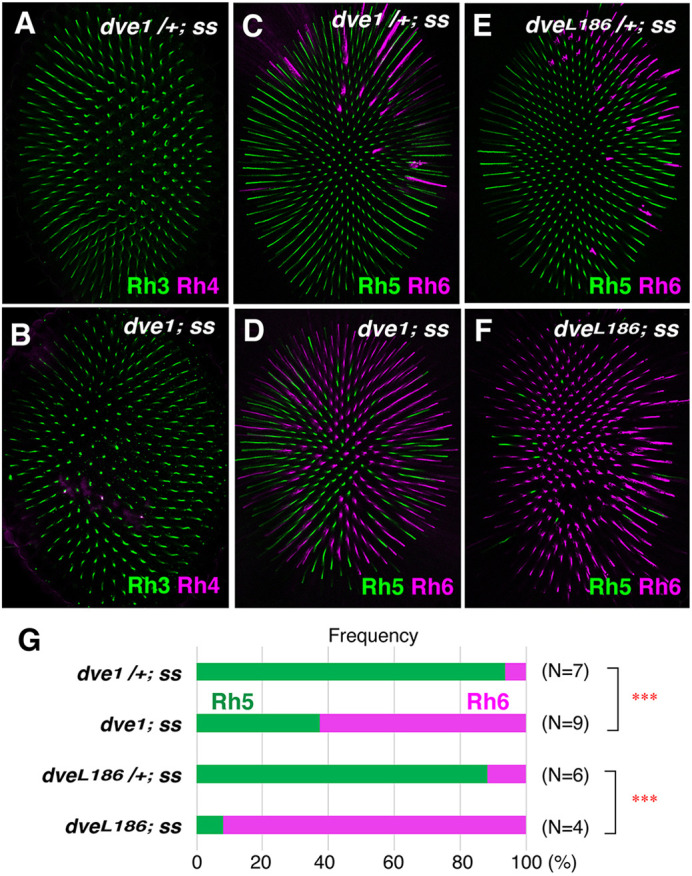
**Dve activity in outer PRs regulates proper coupling.** (A-F) Rhodopsin expression in *ss* mutant eyes [*dve^1^/+; ss^D115.7^* (A,C) and *dve^L186^/+; ss^D115.7^* (E)] and *dve ss* double mutant eyes [*dve^1^; ss^D115.7^* (B,D) and *dve^L186^; ss^D115.7^* (F)]. (A,B) Expression of Rh3 (green) and Rh4 (magenta) in the R7 layer. (C-F) Expression of Rh5 (green) and Rh6 (magenta) in the R8 layer. (G) Expression ratio of the R8 Rhodopsin. *dve^1^/+; ss^D115.7^* (*N*=7 compound eyes, *n*=2929 ommatidia), *dve^1^; ss^D115.7^* (*N*=9, *n*=2242), *dve^L186^/+; ss^D115.7^* (*N*=6, *n*=2515) and *dve^L186^; ss^D115.7^* (*N*=4, *n*=1030). Rh6 is greatly increased in the *dve ss* double mutant background (***P<0.0001, Fisher's exact test).

To further examine functions of Dve for the instructive signal, *dve* mutation was introduced into the *ss* mutant background (*dve ss* double mutants). In *ss* mutant eyes, almost all ommatidia become pale-type coupling (Rh3/Rh5) (Fig. 2A,C,E). Heterozygous or homozygous *dve* mutation in the *ss* mutant background did not affect Rh3 expression in the R7 layer (Fig. 2A,B), whereas *dve ss* double mutant eyes significantly increased the number of Rh6-expressing R8 (Fig. 2D,F,G). These results further support the above notion that Dve activity is essential for transmission of the instructive signal from R7 to R8.

Rhabdomeres of R7 and R8 are vertically aligned on the same axis and their cell bodies are separated by the outer PR, R1, in adult retina (Fig. 1A). However, cell bodies of R7 and R8 appear to contact directly during pupal development (Tomlinson, 1985). Thus, direct transmission of the instructive signal from pale-type R7 (pR7) to R8 is possible, whereas Dve expression is undetectable in these cells. It appears to be unlikely that the undetectable level of Dve expression in pR7 is required for sending the instructive signal from pR7. Therefore, we favor another possibility, that the Dve activity in outer PRs is required for transmission of the instructive signal, because Dve is strongly expressed in outer PRs, R1-R6.

### Dve activity in R1 is required for transmission of the instructive Rh5-inducing signal

**Fig. 3.**
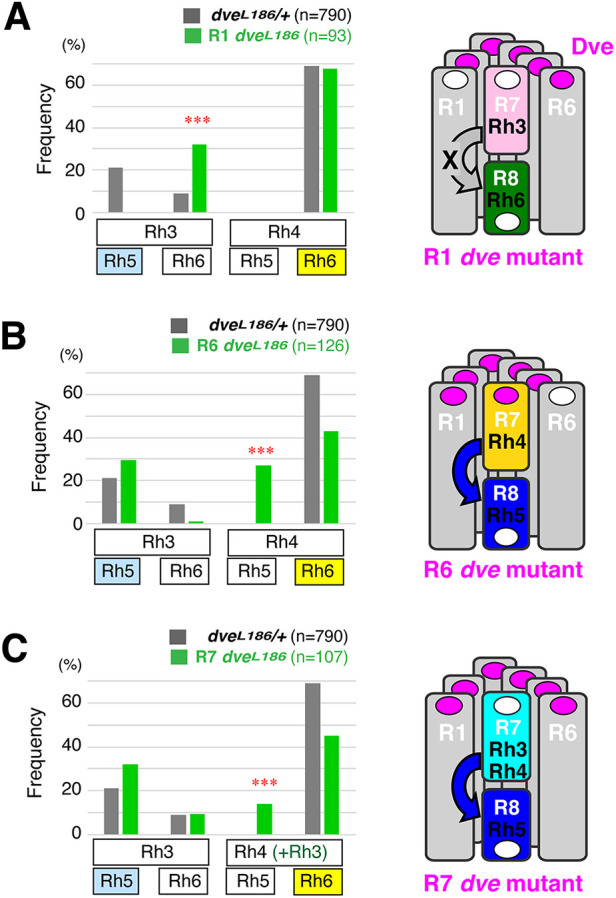
**Two opposite functions of Dve in Rhodopsin coupling.** (A-C) Cell-specific *dve* mutations are induced with the MARCM system. (A) *dve* mutation in R1 (*n*=93). Atypical coupling (Rh3/Rh6) is indicated by asterisks (***P<0.0001, chi-square test). (B) *dve* mutation in R6 (*n*=126). Atypical coupling (Rh4/Rh5) is indicated by asterisks (***P<0.0001, chi-square test). (C) *dve* mutation in R7 (*n*=107). Atypical coupling (Rh3+Rh4/Rh5) is indicated by asterisks (***P<0.0001, chi-square test). Atypical couplings with cell-specific *dve* mutations are schematically shown on the right. Rectangles and circles represent rhabdomeres and nuclei of PR cells, respectively. Dve expression is shown with magenta circles.

Based on the topological arrangement of their cell bodies, we hypothesized that the instructive signal is transmitted from R7 to R1, and then R1 to R8. To induce *dve* mutation in specific cell types, we used *GMR-flp* that expresses Flp recombinase after the second mitotic wave, namely in R1, R6 and R7. In the MARCM system, *dve* mutant cells were labeled as GFP-expressing cells, and a mutant cell completely lost Dve expression (Fig. S1). By using this mosaic system, Rhodopsin coupling was scored in cell-specific *dve* mutant ommatidia (Fig. 3). In R1 *dve* mutant ommatidia, only two types of rhodopsin coupling were observed. These are the default state (Rh3/Rh6) and the yellow type (Rh4/Rh6), and Rh5 was never induced at all. This result clearly shows that the Dve activity in R1 is crucial for transmission of the instructive signal (Fig. 3A).

### Dve activities in R6 and R7 block the instructive Rh5-inducing signal

In R6 or R7 *dve* mutant ommatidia, atypical coupling Rh4/Rh5 was frequently observed (Fig. 3B,C). This coupling was also observed in *dve^1^* mutant eyes (Fig. 1E,F) and reflects ectopic transmission of the instructive signal from Rh4-expressing yR7 to R8. This might be due to ectopic Rh3 expression in yR7. For example, *rh3* knockdown (KD) increased the number of Rh6-expressing R8 to some extent. However, this contribution is redundant, because ectopic transmission was frequently observed with *rh3* KD conditions in R7 *dve* mutants. Furthermore, R6 *dve* mutant ommatidia did not induce ectopic Rh3 expression in yR7, whereas they induced atypical coupling Rh4/Rh5 at 26.9% (Fig. 3B). Thus, ectopic instructive signal can be generated with loss of Dve activities in R6 or R7 rather than the ectopic Rh3 expression in R7.

If the Dve activity in R7 blocks the instructive signal, forced Dve expression in R7 should result in Rh6-expressing R8. As expected, forced Dve expression in all R7 of *ss* mutant eyes substantially blocked the instructive Rh5-inducing signal (Fig. S2). Incomplete inhibition of the instructive signal might be due to the timing of Dve expression, so that the level of GAL4-mediated expression fluctuates among cells. Because GAL4 expression of *panR7-GAL4* is under the control of *rh3* and *rh4* promoters, the signal-sending activity of some R7 appears to be established before a sufficient amount of Dve accumulates. Thus, *dve* loss-of-function and gain-of-function phenotypes are consistent with the notion that the Dve activity in R7 blocks the instructive signal.

**Fig. 4.**
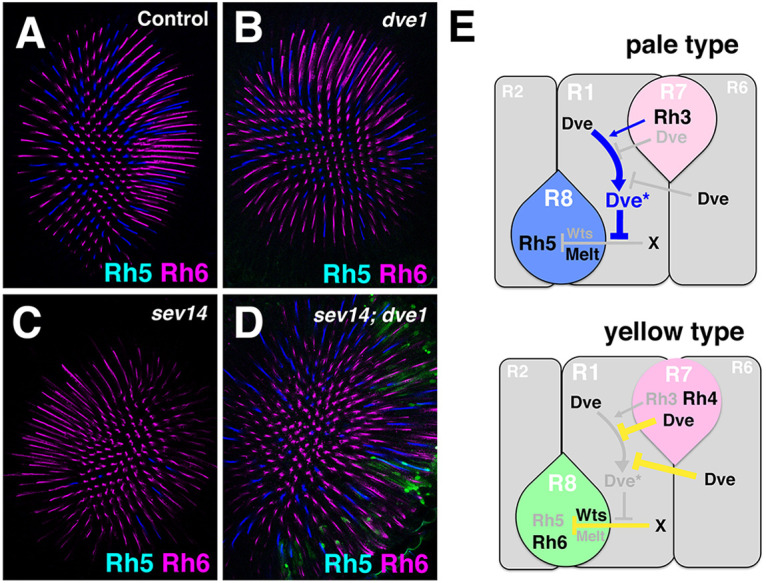
**Rh5-inducing signal can be activated in the absence of R7.** (A-D) Rhodopsin expression in the R8 layer of adult eyes of the indicated genotypes. (A) Control (*sev^14^/w; FRT42D dve^1^/+*)*.* (B) A *dve^1^* mutant eye *(sev^14^/w; FRT42D dve^1^/eyflp FRT42D ubiGFP w^+^M*) shows a nearly normal ratio of Rh5/Rh6 expression (as shown in Fig. 1)*.* (C) A *sev* mutant eye (*sev^14^/Y; FRT42D dve^1^/+*) loses R7 and expresses Rh6 in almost all R8 cells as a default state. Rh5-expressing ommatidia are only 2.5% (*N*=6 compound eyes, *n*=1397 ommatidia). (D) A *sev dve* double mutant eye (*sev^14^/Y; FRT42D dve^1^/eyflp FRT42D ubiGFP w^+^M*) expresses Rh5 in 22% ommatidia (*N*=10, *n*=2419; chi-square test, *P*<0.0001). Almost all cells that do not express GFP (green) are *dve^1^* mutant. Rh5 (blue) and Rh6 (magenta) are shown. (E) Rh5 expression in the R8 is repressed by Hippo signaling (Wts/Melt) through unknown signal ‘X’. In pale-type ommatidia, Rh3-expressing R7 sends a signal to the adjacent R1. The activated form of Dve (Dve*) in the R1 represses the signal ‘X’ and induces Rh5 expression through a relief-of-repression mechanism. In yellow-type ommatidia, Spineless (Ss) induces Rh4 and Dve expression in the R7, and Dve represses Rh3 expression (Johnston et al., 2011). Dve activities in the R7 and R6 repress the activation of Dve in the R1 and lead to a default state of Rh6 expression.

To further examine the mechanism, we used a *sevenless* (*sev^14^*) mutant that loses R7. In the R8 layer of *sev^14^* mutant eyes, almost all ommatidia express Rh6 as a default state (Fig. 4A-C). In *sev^14^ dve^1^* double mutant eyes, Rh5-expressing ommatidia were significantly increased (Fig. 4D), indicating that the instructive signal can be generated in the absence of R7. Taken together, these results strongly suggest that the Dve activities in R6 and R7 are required to block the instructive Rh5-inducing signal by affecting functions of adjacent R1.

### Mechanisms of Rhodopsin coupling

In human retinas, the orphan nuclear receptor NR2E3 (also known as PNR) is expressed in rod cells (Bumsted O'Brien et al., 2004; Cheng et al., 2004) and activates the expression of rod genes but represses that of cone genes (Peng et al., 2005). Interestingly, mutations in the NR2E3 gene not only cause defects in the rod system but also change the sensitivity of cone cells. NR2E3 mutant retinas are hypersensitive to blue light (S-cone) and have reduced sensitivity to green and red light (M- and L-cones), leading to ‘enhanced S-cone syndrome’ (Haider et al., 2000). The NR2E3 mutant phenotype is similar to that of our observation, because *dve* mutation in R1 or R6 affects Rhodopsin expression in R8. Although loss of function in rod cells affects functions in cone cells, it is due to misdifferentiation of rod precursor cells into the default cell type, S-cones, but not through abnormal intercellular signaling (Cheng et al., 2011).

Our results provide first evidence that functional opsin patterning (in R7/R8) for *Drosophila* color vision is established through signaling pathways in adjacent object-detection neurons (R1 and R6). Our model is shown in Fig. 4E. In a default state, Rh5 expression is repressed by the Hippo pathway in R8. In pale-type ommatidia, Rh3 expression in R7 sends an instructive Rh5-inducing signal and relieves repression of Rh5 in R8 through disruption of the Hippo signaling pathway (Mikeladze-Dvali et al., 2005). Our results suggest that the instructive signal from R7 activates Dve in adjacent R1 presumably through post-translational modification, and that the activated Dve (Dve*) disrupts the Hippo signaling pathway in adjacent R8. In yellow type ommatidia, Dve activities in R7 and R6 appear to repress the activation of Dve in R1, resulting in active Hippo signaling and Rh6 expression in R8. Activin and BMP signaling are required upstream of the Hippo pathway to establish expression of Rh5 or Rh6 in R8 (Wells et al., 2017). If R7 secretes Activin and BMP ligands, these pathways might be independent of the Dve-mediated pathway because the instructive Rh5-inducing signal can be activated in the absence of R7 (Fig. 4).

As Dve represses Rh3, Rh5 and Rh6 in outer PR cells (Johnston et al., 2011), another possible mechanism is that Dve represses the instructive signal as a default state of outer PRs. In this case, the signal from R7 inhibits Dve in outer PRs and induces a relief of repression to induce Rh5. However, this is inconsistent with the result that R1 *dve* mutant completely blocks the signal. Thus, we propose a model whereby Dve* in R1 is involved in the instructive Rh5-inducing signal (Fig. 4E).

In mouse retinas, thyroid hormone (TH) and TH receptor β2 is required to activate M-opsin and to repress S-opsin (Glaschke et al., 2011; Ng et al., 2001; Roberts et al., 2006). These reports suggest that extrinsic signals are required for spatial distribution and their subtype specification of cone cells. Moreover, our results raise an intriguing possibility that local intercellular signaling between rod and cone cells is also important for subtype specification. Thus, further characterization of *Drosophila* color vision will provide insights into the mechanism of functional specification during retinal development.

## MATERIALS AND METHODS

### *Drosophila* strains

All flies were reared on a standard yeast and cornmeal-based diet at 25°C. Oregon-R (OR) flies were used as wild-type controls. *dve^1^* is a severe loss-of-function allele that has no *dve-A* and a very weak *dve-B* activity in the larval midgut (Nakagawa et al., 2011; Nakagoshi et al., 1998). *dve^L186^* and *ss^D115.7^* are null alleles (Duncan et al., 1998; Terriente et al., 2008). *UAS-dveA-9A4* has been described previously (Nakagawa et al., 2011). The following lines were used from the Bloomington Drosophila Stock Center: *w^1118^ sev^14^* (10546) and *panR7-GAL4* (8603), and FLP lines that express FLP in the eye-antennal disc (*ey-flp2*, 5580) and in R1, R6 and R7 (*GMR-flp*, 42735). *GMR-wIR* (gift from R. Carthew, Northwestern University, Evanston, IL, USA) was used to induce *white* RNAi in adult eyes (Lee and Carthew, 2003).

### Mosaic analyses

Mutant mosaic clones were induced by the FRT- and FLP-mediated recombination system (Xu and Rubin, 1993) as the following genotypes:

*yw ey-flp2/Y; FRT42D dve^1^/FRT42D w^+^ M(2)53^1^*

*yw ey-flp2/Y; FRT42D dve^L186^/FRT42D w^+^ M(2)53^1^*

yw ey-flp2/Y; FRT42D dve^1^/+; FRT82B ss^D115.7^/FRT82B w^+^ l(3)cl-R3

*yw ey-flp2/Y; FRT42D dve^1^/FRT42 GMR-hid; FRT82B ss^D115.7^/FRT82B w^+^ l(3)cl-R3*

*yw eyflp2/GMR-wIR; FRT42D dve^L186^/+; FRT82B ss^D115.7^/FRT82B GMR-hid l(3)CL-R1*

*yw eyflp2/GMR-wIR; FRT42D dve^L186^/FRT42 GMR-hid; FRT82B ss^D115.7^/FRT82B GMR-hid l(3)CL-R1*

*yw eyflp2/Y; panR7-GAL4/UAS-dveA-9A4; FRT82B ss^D115.7^/ FRT82B w^+^ l(3)cl-R3*.

Mutant mosaic clones labeled with GFP were induced by the MARCM system (Lee and Luo, 1999) as the following genotypes:

*GMR-flp yw/UAS-ActGFP; FRTG13 UAS-mCD8-GFP dve¹/FRTG13 tub-GAL80; tub-GAL4/GMR-wIR*

*GMR-flp yw/UAS-ActGFP; FRTG13 UAS-mCD8-GFP dve^L186^/FRTG13 tub-GAL80; tub-GAL4/GMR-wIR*.

### Immunohistochemistry

Adult compound eyes were dissected in phosphate-buffered saline (PBS), fixed with 4% formaldehyde/PBS-0.3% Triton X-100 for 15 min, and washed three times with PBS-0.3% Triton X-100. The following primary antibodies were used: rabbit anti-Dve (1:2000; Nakagoshi et al., 1998), mouse anti-Rh3 (1:20, 2B1, gift from S. Britt, University of Texas at Austin, USA), rabbit anti-Rh4 (1:200, gift from C. S. Zuker, Columbia University, New York, USA), mouse anti-Rh5 (1:200, 7F1, gift from S. Britt), rabbit anti-Rh6 (1:2000, gift from C. Desplan, New York University, USA). FITC-, Cy3- or Cy5-conjugated secondary antibodies (115-095-146, 115-165-146, 111-165-144 and 111-175-144, Jackson ImmunoResearch) were used for detection. Phalloidin-TRITC (Sigma-Aldrich) was used to stain actin fibers of rhabdomere. Confocal images of 0.2-1.22 µm sections were obtained using a confocal microscope (Olympus FV1200) and were processed using the Fluoview (Olympus) and Photoshop (Adobe) software.

### Statistical analysis

The significance of differences between the control and test progenies was analyzed using Prism6 (GraphPad Software). A chi-square test was applied to compare the frequency distribution of Rhodopsin pairing (ommatidial subtypes). Fisher's exact test was applied to compare the frequency distribution of Rhodopsin expression in R8. The levels of significance are indicated by asterisks: *P<0.01, **P<0.001, ***P<0.0001.

## Supplementary Material



10.1242/develop.202388_sup1 Supplementary information
