# Supplementary material for: Functional opsin patterning for Drosophila color vision is established through signaling pathways in adjacent object-detection neurons
Source: Development. 2024 Mar 15;151(6):dev202388. doi: 10.1242/dev.202388 (PMC10984275; doi:10.1242/dev.202388)
Supplement: Supplementary information [file develop-151-202388-s1.pdf]

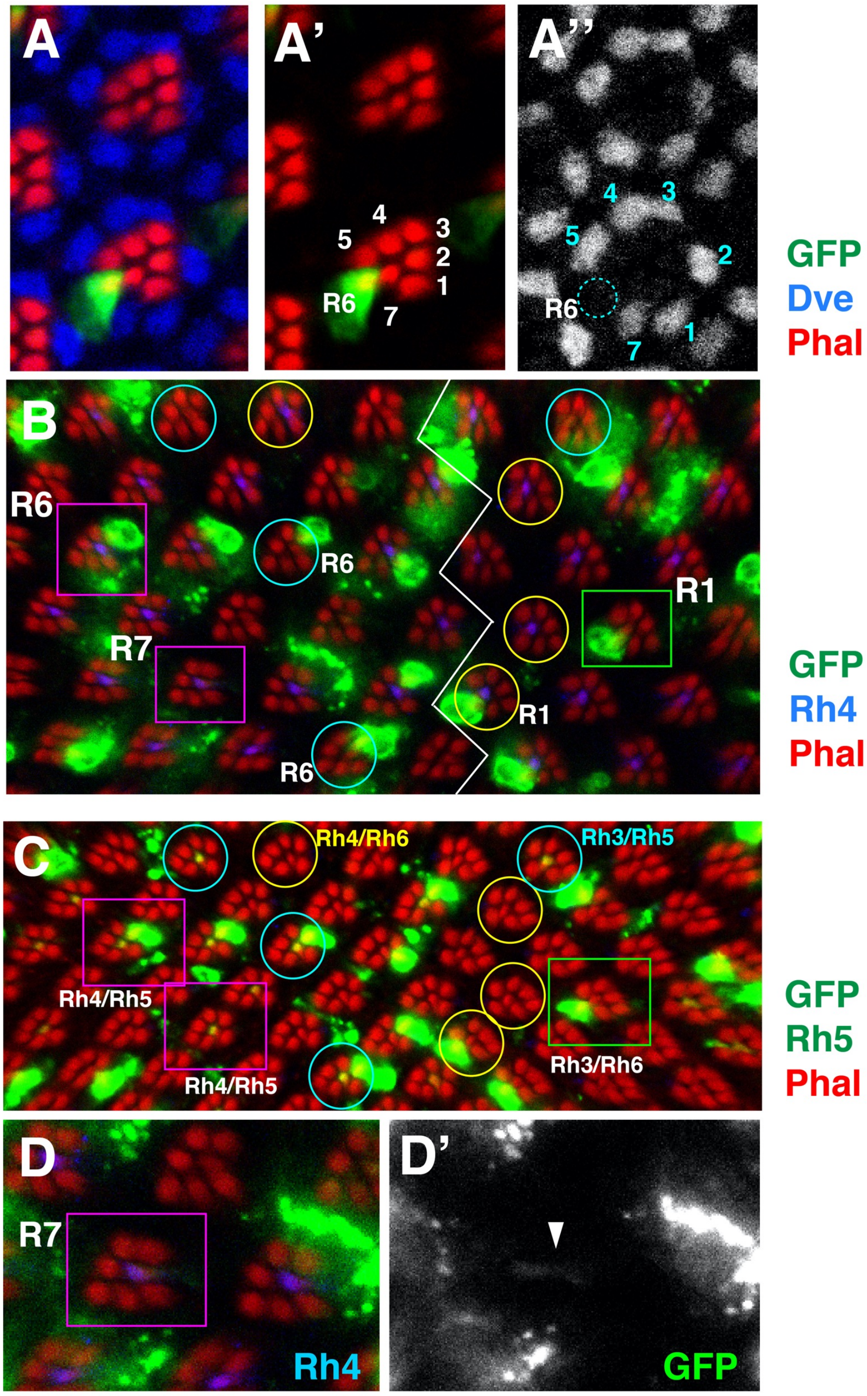

Fig. S1. Cell-specific *dve* mutant clones by the MARCM system

Cell-specific *dve* mutations are labeled with GFP expression (green) in the MARCM system (*GMR-flp/UAS-actGFP; FRTG13 GAL80/FRTG13 UAS-mCD8-GFP dve^{L186}; tub-GAL4/GMR-wIR*). Rhabdomeres are labeled with Phalloidin (red). (A) An ommatidium with R6-specific *dve* mutation. Dve (blue). The position of R1-R7 is numbered in A' and A''. Loss of Dve expression in R6 is indicated as a dotted circle in A''. (B-D) *dve* mutant ommatidia in the R7 layer (B) and the R8 layer (C). Rh4 (blue) and Rh5 (green) are shown. Ommatidia with typical Rh couplings are outlined with circles (pale and yellow, Rh3/Rh5 and Rh4/Rh6, respectively). Atypical types of ommatidia are outlined with squares (green and magenta, Rh3/Rh6 and Rh4/Rh5, respectively). Mutant R cells (R1, R6, and R7) and the dorso-ventral boundary of the equator (white line) are shown in (B) and the Rh coupling is shown in (C). (D) A magnified view of an ommatidium with R7-specific *dve* mutation in (B). (D') GFP expression in the R7 rhabdomere is indicated by an arrowhead.

Development • Supplementary information

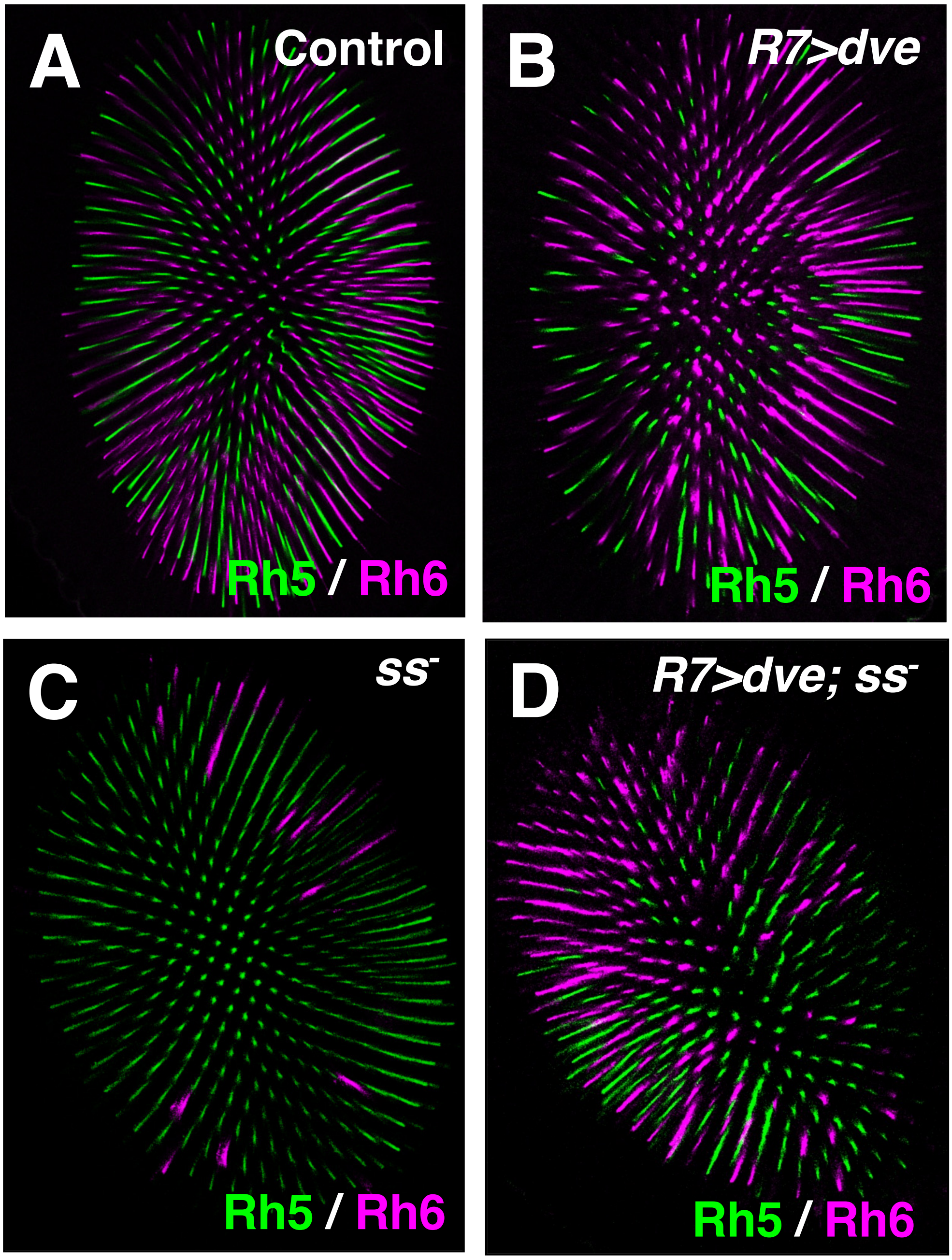

Fig. S2. Forced Dve expression in R7 blocks the Rh5-inducing signal

Rhodopsin expression in the R8 layer of adult eyes of the indicated genotypes. (A) Control (*UAS-dveA-9A4/CyOGFP; +/GMR-wIR),* (B) Forced *dve* expression in R7 *(panR7-GAL4/UAS-dveA-9A4; +/GMR-wIR),* (C) *ss* mutant (*yw eyflp2/Y; UAS-dveA-9A4/CyOGFP; FRT82B* $ss^{D115.7}$*/FRT82B* w^{+} *M*), and (D) Forced *dve* expression in R7 of *ss* mutant (*yw eyflp2/Y; panR7-GAL4/UAS-dveA-9A4; FRT82B* $ss^{D115.7}$*/ FRT82B* w^{+} *M*). Rh5 (green) and Rh6 (magenta) are shown.

Development • Supplementary information
