# Supplementary material for: Functional opsin patterning for Drosophila color vision is established through signaling pathways in adjacent object-detection neurons
Source: Development. 2024 Mar 15;151(6):dev202388. doi: 10.1242/dev.202388 (PMC10984275; doi:10.1242/dev.202388)
Supplement: Supplementary file 1 [file develop-151-202388_review_history.pdf]

Functional opsin patterning for *Drosophila* color vision is established through signaling pathways in adjacent object-detection neurons

Manabu Kitamata, Yoshiaki Otake, Hideaki Kitagori, Xuanshuo Zhang, Yusuke Maki, Rika Boku, Masato Takeuchi and Hideki Nakagoshi
DOI: 10.1242/dev.202388

Editor: Francois Guillemot

Review timeline
Original submission: 29 September 2023
Editorial decision: 22 November 2023
First revision received: 26 January 2024
Accepted: 22 February 2024

Original submission

First decision letter

MS ID#: DEVELOP/2023/202388

MS TITLE: Functional opsin patterning for Drosophila color vision is established through signaling pathways in adjacent object-detection neurons

AUTHORS: Manabu Kitamata, Yoshiaki Otake, Hideaki Kitagori, Xuanshuo Zhang, Yusuke Maki, Rika Boku, Masato Takeuchi, and Hideki Nakagoshi

I have now received the reports of three referees on your manuscript and I have reached a decision. The referees' comments are appended below, or you can access them online: please go to BenchPressand click on the 'Manuscripts with Decisions' queue in the Author Area.

As you will see, all the referees express great interest in your work, but they also have significant criticisms and recommend a substantial revision of your manuscript before we can consider publication. If you are able to revise the manuscript along the lines suggested, which may involve further experiments, I will be happy to receive a revised version of the manuscript. You will see that referee 2 is more critical and has a number of requests, some of which seem beyond the scope of the paper, including point 1 on the identification of a non-autonomous signal, point 2 on the identification of the activated Dve protein, point 3 on data from mutant R2, R3, R4 and R5 cells, and point 5 on an over-expression Dve phenotype unless this has already been done in published articles. I do not request that you address experimentally these particular points but you can of course comment on them in the manuscript.

Your revised paper will be re-reviewed by the original referees, and its acceptance will depend on your addressing satisfactorily all their major concerns. Please also note that Development will normally permit only one round of major revision. If it would be helpful, you are welcome to contact us to discuss your revision in greater detail. Please send us a point-by-point response indicating your plans for addressing the referees' comments, and we will look over this and provide further guidance.

Please attend to all of the reviewers' comments and ensure that you clearly highlight all changes made in the revised manuscript. Please avoid using 'Tracked changes' in Word files as these are lost

© 2024. Published by The Company of Biologists under the terms of the Creative Commons Attribution License (https://creativecommons.org/licenses/by/4.0/).

in PDF conversion. I should be grateful if you would also provide a point-by-point response detailing how you have dealt with the points raised by the reviewers in the 'Response to Reviewers' box. If you do not agree with any of their criticisms or suggestions please explain clearly why this is so.

Reviewer 1

Advance summary and potential significance to field

The manuscript by Kitamata and colleagues describes and interesting mechanism by which coupling of rhodopsin genes in adjacent photoreceptor cells is mediated by the transcription factor defective proventriculus (dve). It involves complex signaling between several cells. The R7 and R8 cells of the ommatidium are the ones in which rhodopsin genes are coupled. The activity of Dve in the R7 and two adjacent cells R1 and R6 appear to be involved. The authors model invokes signaling from the R1 and the R6 cell and the distinction between two different versions of Dve – an active form and an inactive version. This manuscript has the potential to provide insight into how rhodopsin genes are coupled in fly photoreceptors.
This could have implications for how rhodopsins are expressed and coupled in vertebrate retinas.

Comments for the author

The manuscript by Kitamata and colleagues describes and interesting mechanism by which coupling of rhodopsin genes in adjacent photoreceptor cells is mediated by the transcription factor defective proventriculus (dve). It involves complex signaling between several cells. The R7 and R8 cells of the ommatidium are the ones in which rhodopsin genes are coupled. The activity of Dve in the R7 and two adjacent cells R1 and R6 appear to be involved. The authors model invokes signaling from the R1 and the R6 cell and the distinction between two different versions of Dve – an active form and an inactive version. This is an interesting manuscript, but I am concerned that it is at a preliminary stage. Overall, the results are interesting but there are just too many unknowns within the model for the manuscript to be published in Development at this point.

I have some suggestions for the authors.

1. The manuscript would be significantly strengthened if the authors can identify at least one of the non-autonomous signals that are proposed to be functioning in the R1 and R6 cells.

2. Similarly, the manuscript and model would be stronger if the authors could identify the nature of the activated Dve protein that they predict to exist in the R1 photoreceptor.

3. It is interesting that only the R1 and R6 photoreceptor neurons are the only ones involved. Please include and comment on the data for the mutant R2, R3, R4, and R5 cells. This could be included in the supplemental information but commented on in the main text.

4. The authors mention that the R1 photoreceptor separates the R7 and R6 cell bodies. Could they include a reference for that piece of information.

5. It would be interesting to compare the loss of function dve phenotype with an over-expression dve phenotype.

Reviewer 2

Advance summary and potential significance to field

See attached

Comments for the author

Review of Kitamata et al 2023
In this paper, Kitamata and colleagues investigate the role of the transcription factor Dve in

© 2024. Published by The Company of Biologists under the terms of the Creative Commons Attribution License (https://creativecommons.org/licenses/by/4.0/).

photoreceptor patterning in the fly eye, specifically how it regulates the coordination of photoreceptor subtype fates. The fly eye is comprised of six outer photoreceptors (R1-R6) and two inner photoreceptors (R7 and R8). All outer photoreceptors express Rhodopsin1 (Rh1). In contrast, there are two subtypes of R7s and R8s defined by expression of different Rh proteins. Pale R7s expressing Rh3 are coupled with pale R8s expressing Rh5. Yellow R7s expressing Rh4 are coupled with yellow R8s expressing Rh6. The pale vs yellow cell fate decision is made in R7s and signaling from R7s to R8s coordinates these cell fates. The authors showed previously that Dve is expressed in all outer photoreceptors (R1-R6) and yellow R7s. In this paper, the authors describe the role of Dve in regulating the signaling between R7s and R8s. They find that *dve* mutants display miscoordination between R7 and R8 subtypes. They show that Dve is required in R1 photoreceptors for correct pR7/Rh3 to pR8/Rh5 fate coordination, as single cell mutant clones yield pR7/Rh3 to yR8/Rh6 miscoordination. They show that Dve plays a role in R6 and R7 photoreceptors as single cell mutant clones yield yR7/Rh4 to pR8/Rh5 miscoordination.
This work shows interesting observations, and the results suggest two main conclusions: 1. Dve plays specific roles in the coordination of photoreceptor subtype fates, and 2. Outer photoreceptors play roles in this process. The data are clear and mostly convincing. The biggest weaknesses of the paper are that the authors significantly overinterpret their results and overstate conclusions. The authors must provide alternative interpretations/conclusions **OR** additional experiments to support their main conclusion. I suggest that major text edits and minor changes in data presentation will improve this paper for publication.

Major comments

1. **Overinterpretation of results should be corrected:** The authors clearly show that Dve mutant clones in R1 result in pR7/Rh3 to yR8/Rh6 miscoordination and that Dve mutant clones in R7 and R6 result in yR7/Rh4 to pR8/Rh5 miscoordination. As stated above, these data show that 1. Dve plays specific roles in the coordination of photoreceptor subtype fates, and 2. Outer photoreceptors play roles in this process. These findings and conclusions are significant.
The authors make numerous overstatements about these results. Here is one example:
If the instructive signal is directly transmitted from pR7 to R8, the signal transduction between rhabdomeres is probable, because their cell bodies are separated by the outer PR, R1. Lines 118-126

Another example is
Rhabdomeres of R7 and R8 are vertically aligned on the same axis but their cell bodies are separated by the outer PR, R1.
There are several more cases of these types of overstatement. These types of statements are found in the results section and stated as if the interpretation is conclusive. These types of statements should be toned down and included in the discussion as possible mechanisms to explain their observations.

2. **Alternative interpretations should be included:** The authors only explore their single interpretation of their data. However, without additional experiments (which are otherwise not needed for this paper), they should provide alternative ideas about mechanisms driving these processes. These include:

a. **Timing:** The timing of the role of Dve could be important. How does the timing of Dve in outer PRs and R7s match up to the signaling event? Does Dve likely play a role in establishment and/or maintenance of signaling?

b. ***dve* mutant phenotypes could simply perturb coordination by derepression of signaling:** The authors conclude that Dve in outer PRs plays a direct role in this process. The authors showed previously that Dve represses inner PR fate in the outers by repressing Rh3, Rh5, Rh6. In the outer PRs, Dve could simply be repressing signals and *dve* mutants yield derepression of these signals to perturb coordination of fates.
To support the conclusion of the authors, they would have to ablate the cells and/or convert their fates and look at coordination, or determine what genes Dve regulates in outer PRs to control coordination. The authors should either provide these experiments **OR** provide alternative interpretations of their current data.

3. **The method to assess coordination is not clearly presented:** In figure 3, the authors provide the data about coordination. However, they do not clearly describe how they did these experiments in terms of assessing cell fates. They authors should 1. provide a clear description of how they did the imaging and quantification and 2. Provide example images for all relevant phenotypes for all types of single cell clones.

© 2024. Published by The Company of Biologists under the terms of the Creative Commons Attribution License (https://creativecommons.org/licenses/by/4.0/).

Minor comments
1. The paper would benefit from an introduction figure to outline the ideas to the reader.
2. The retinas should be rotated 90° and shown with dorsal at the top.

Reviewer 3

Advance summary and potential significance to field

Sensory systems often rely on stochastic choices to express one sensory receptor gene out of many into each of the sensory neurons. The Drosophila fly eye serves as a particularly beneficial system to study the molecular events involved in stochastic choices because of its extreme simplicity (a binary stochastic choice), the deep knowledge of the system and the many tools available to study its molecular genetics.
This paper takes advantage of this to propose a highly unexpected model that adds a surprising level of complexity to this apparently extremely simple system.
The choice in R7 cells to express Rh4 vs Rh3 is driven by the stochastic expression of spineless (ss) in Rh4-expressing cells. These cells repress Rh3 because Ss turns on another transcription factor, Dve in these cells and Dve represses Rh3. This is well established and a role of Dve restricted to repressing Rh3.
Where things become less clear is how R7 instructs the R8 cell below it to express Rh5 if R7 expresses Rh3 and Rh6 if R7 expresses Rh4. The latter is the default state since, in the total absence of R7 (sevenless), (almost) all R8 become Rh6.
The issue investigated here is why not only Rh4-R7 express Dve, but all outer photoreceptors R1-R6 also do express it.
Furthermore, the authors show that, in a null dve, ss double mutant, in which all R7 express Rh3 (and no dve), most R8 express Rh6: This means that Dve is involved in transmitting the signal from R7 to R8, but not by virtue of its expression in R7 since it is not expressed in the Rh3-R7.
The authors thus perform a clever experiment by benefiting from an old trick that allows one to mutate individually R7, R1 or R6, the last three cells born before the last mitosis.
The results are surprising:
- R1 dve mutants reproduce the whole eye null dve mutant: all R8 become Rh6 showing that the signal is conveyed by Dve in R1. How? This remains to be investigated
- More surprisingly, when R6 is mutant, now Rh5 is expressed in (many, not all)
R8 below Rh4-R7 cells, suggesting that a signal mediated by R6 represses the Rh5 fate in ss ommatidia.
- Finally, when R7 is mutant for Dve in Rh4-R7, which now co-express Rh3 and Rh4 because Rh3 is no longer repressed, Rh5 is also coupled with Rh4

The conclusion is surprising as it shows that there is both a signal from Rh3-R7 to Rh5-R8 but there is also a signal to repress Rh5 in ommatidia that express ss. And both are mediated by Dve in R1, or in R6/R7!

This is a striking result that should be published.

Comments for the author

This is a striking result that should be published.

I simply would like to suggest a few things:
- The paper has only three figures and a model. The authors must show several examples of R1, R6 and R7 mutant ommatidia instead than just the table of results: These experiments are often difficult to read and require a very good marking of the mutant cells. This would show that the data are very solid.
- In the discussion, the authors mention “activation of Dve”, which in one instance they correctly label Dve*. They should make sure that the readers understand that this is not transcriptional. In fact, Dve might be permissive here and simply allow the signal to be transmitted (or not) by R1 or R6.

© 2024. Published by The Company of Biologists under the terms of the Creative Commons Attribution License (https://creativecommons.org/licenses/by/4.0/).

- There were attempts to identify the signal from Rh3 to Rh5, which involves the BMP/Activin pathways, but this was difficult to interpret. The authors should revisit these data in view of their model in the discussion.
- The paper is at time difficult to read for a non-specialist due to the confusion of nomenclature, R1, Rh3, Rh6, R6, R7...... The authors should make even more efforts to make these things clear

---

First revision

Author response to reviewers' comments

Responses to Reviewers' comments

Reviewers' comments are colored in blue.
Our responses are listed below.

MS ID#: DEVELOP/2023/202388
MS TITLE: Functional opsin patterning for Drosophila color vision is established through signaling pathways in adjacent object-detection neurons
AUTHORS: Manabu Kitamata, Yoshiaki Otake, Hideaki Kitagori, Xuanshuo Zhang, Yusuke Maki, Rika Boku, Masato Takeuchi, and Hideki Nakagoshi
----

Reviewer 1 Advance Summary and Potential Significance to Field:
The manuscript by Kitamata and colleagues describes and interesting mechanism by which coupling of rhodopsin genes in adjacent photoreceptor cells is mediated by the transcription factor defective proventriculus (dve). It involves complex signaling between several cells. The R7 and R8 cells of the ommatidium are the ones in which rhodopsin genes are coupled. The activity of Dve in the R7 and two adjacent cells R1 and R6 appear to be involved. The authors model invokes signaling from the R1 and the R6 cell and the distinction between two different versions of Dve - an active form and an inactive version. This manuscript has the potential to provide insight into how rhodopsin genes are coupled in fly photoreceptors.
This could have implications for how rhodopsins are expressed and coupled in vertebrate retinas.
Reviewer 1 Comments for the Author:
The manuscript by Kitamata and colleagues describes and interesting mechanism by which coupling of rhodopsin genes in adjacent photoreceptor cells is mediated by the transcription factor defective proventriculus (dve). It involves complex signaling between several cells. The R7 and R8 cells of the ommatidium are the ones in which rhodopsin genes are coupled. The activity of Dve in the R7 and two adjacent cells R1 and R6 appear to be involved. The authors model invokes signaling from the R1 and the R6 cell and the distinction between two different versions of Dve - an active form and an inactive version. This is an interesting manuscript, but I am concerned that it is at a preliminary stage. Overall, the results are interesting but there are just too many unknowns within the model for the manuscript to be published in Development at this point.
I have some suggestions for the authors.
1. The manuscript would be significantly strengthened if the authors can identify at least one of the nonautonomous signals that are proposed to be functioning in the R1 and R6 cells.
2. Similarly, the manuscript and model would be stronger if the authors could identify the nature of the activated Dve protein that they predict to exist in the R1 photoreceptor.
Points 1 and 2 are very important and we also want to know, but it seems to be very difficult. Our preliminary results suggest that several cell junction molecules are involved in transmission of the instructive Rh5-inducing signal. However, we need further experiments to get solid results, and these results seem to be beyond the scope of this work. "Research reports" in Development has a word limitation less than 3000 words. So, we think that we should focus on the Dve

© 2024. Published by The Company of Biologists under the terms of the Creative Commons Attribution License (https://creativecommons.org/licenses/by/4.0/).

activities required in outer photoreceptor cells.

3. It is interesting that only the R1 and R6 photoreceptor neurons are the only ones involved. Please include and comment on the data for the mutant R2, R3, R4, and R5 cells. This could be included in the supplemental information but commented on in the main text.

Before using *GMR-flp*, we carried out outer cell specific *dve* mutant analyses by using MARCM system with *eyeless-flp* and *rh1-GAL4*, which expresses GAL4 in R1-R6. Although there was experimental difficulty that single cell mutants were rarely recovered, Dve activities in R1 and R6 were thought to be important for the instructive signal. Based on these results, we focused our analyses on R1 and R6 using *GMR-flp*.
NOTE: We have removed unpublished data that had been provided for the referees in confidence.

4. The authors mention that the R1 photoreceptor separates the R7 and R6 cell bodies. Could they include a reference for that piece of information.

We added Fig. 1A for an introduction figure and cited a paper (Tomlinson, 1985) as follows.
Rhabdomeres of R7 and R8 are vertically aligned on the same axis and their cell bodies are separated by the outer PR, R1, in adult retina (Fig. 1A). However, cell bodies of R7 and R8 appear to contact directly during pupal development (Tomlinson, 1985).

Tomlinson, A. (1985) The cellular dynamics of pattern formation in the eye of *Drosophila*. Journal of Embryology and Experimental Morphology 89, 313-331.

NOTE: Figure provided for reviewer has been removed. It showed Figure 1 from Tomlinson, A. (1985) The cellular dynamics of pattern formation in the eye of *Drosophila*. *J Embryol Exp Morphol*. 89, 313-331. doi:10.1242/dev.89.1.313

Fig. 1 (A) Schematic representation of rhabdomere arrangement of two major ommatidial subtypes (pale and yellow). Rectangles and circles represent rhabdomeres and nuclei of photoreceptor cells (R1-R8 in white characters), respectively. Rhodopsin expression (Rh1, Rh3-Rh6) in rhabdomeres is shown in differently colored rectangles. Dve expression in nuclei is shown with magenta circles. The instructive Rh5-inducing signal from R7 to R8 is shown by a curved arrow. Horizontal sections of R7 and R8 layers show the position of rhabdomeres and cell bodies.

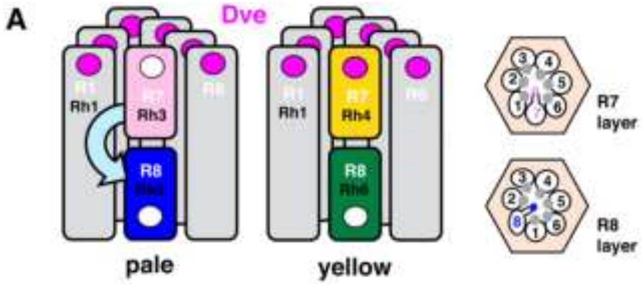

5. It would be interesting to compare the loss of function dve phenotype with an over-expression dve phenotype.

We added Dve over-expression experiments (Fig. S2) and *sev dve* double mutant phenotype (Fig. 4A-D), and added the description as follows.

© 2024. Published by The Company of Biologists under the terms of the Creative Commons Attribution License (https://creativecommons.org/licenses/by/4.0/).

If the Dve activity in R7 blocks the instructive signal, forced Dve expression in R7 should result in Rh6-expressing R8. As expected, forced Dve expression in all R7 of *ss* mutant eyes substantially blocked the instructive Rh5-inducing signal (Fig. S2). Incomplete inhibition of the instructive signal might be due to the timing of Dve expression, that the level of GAL4-mediated expression fluctuates among cells. Because GAL4 expression of *panR7-GAL4* is under the control of *rh3* and *rh4* promoters, the signal-sending activity of some R7 appears to be established before sufficient amount of Dve accumulates. Thus, *dve* loss-of-function and gain-of-function phenotypes are consistent with the notion that the Dve activity in R7 blocks the instructive signal.

To further examine the mechanism, we used *sevenless* (*sev*^{14}) mutant that loses R7. In the R8 layer of *sev*^{14} mutant eyes, almost all ommatidia express Rh6 as a default state (Fig. 4A-C). In *sev*^{14} *dve*^{1} double mutant eyes, Rh5-expressing ommatidia were significantly increased (Fig. 4D), indicating that the instructive signal can be generated in the absence of R7. Taken together, these results indicate that the Dve activities in R6 and R7 are required to block the instructive Rh5- inducing signal by affecting functions of adjacent R1.

***** Reviewer 2 Comments for the Author:

In this paper, Kitamata and colleagues investigate the role of the transcription factor Dve in photoreceptor patterning in the fly eye, specifically how it regulates the coordination of photoreceptor subtype fates. The fly eye is comprised of six outer photoreceptors (R1-R6) and two inner photoreceptors (R7 and R8). All outer photoreceptors express Rhodopsin1 (Rh1). In contrast, there are two subtypes of R7s and R8s defined by expression of different Rh proteins. Pale R7s expressing Rh3 are coupled with pale R8s expressing Rh5. Yellow R7s expressing Rh4 are coupled with yellow R8s expressing Rh6. The pale vs yellow cell fate decision is made in R7s and signaling from R7s to R8s coordinates these cell fates. The authors showed previously that Dve is expressed in all outer photoreceptors (R1-R6) and yellow R7s. In this paper, the authors describe the role of Dve in regulating the signaling between R7s and R8s. They find that dve mutants display miscoordination between R7 and R8 subtypes. They show that Dve is required in R1 photoreceptors for correct pR7/Rh3 to pR8/Rh5 fate coordination, as single cell mutant clones yield pR7/Rh3 to yR8/Rh6 miscoordination. They show that Dve plays a role in R6 and R7 photoreceptors as single cell mutant clones yield yR7/Rh4 to pR8/Rh5 miscoordination.

This work shows interesting observations, and the results suggest two main conclusions: 1. Dve plays specific roles in the coordination of photoreceptor subtype fates, and 2. Outer photoreceptors play roles in this process. The data are clear and mostly convincing. The biggest weaknesses of the paper are that the authors significantly overinterpret their results and overstate conclusions. The authors must provide alternative interpretations/conclusions OR additional experiments to support their main conclusion. I suggest that major text edits and minor changes in data presentation will improve this paper for publication.

Major comments

1. Overinterpretation of results should be corrected: The authors clearly show that Dve mutant clones in R1 result in pR7/Rh3 to yR8/Rh6 miscoordination and that Dve mutant clones in R7 and R6 result in yR7/Rh4 to pR8/Rh5 miscoordination. As stated above, these data show that 1. Dve plays specific roles in the coordination of photoreceptor subtype fates, and 2. Outer photoreceptors play roles in this process. These findings and conclusions are significant.

The authors make numerous overstatements about these results. Here is one example:

If the instructive signal is directly transmitted from pR7 to R8, the signal transduction between rhabdomeres is probable, because their cell bodies are separated by the outer PR, R1.

Lines 118-126

Another example is

Rhabdomeres of R7 and R8 are vertically aligned on the same axis but their cell bodies are separated by the outer PR, R1.

There are several more cases of these types of overstatement. These types of statements are found in the results section and stated as if the interpretation is conclusive. These types of statements should be toned down and included in the

© 2024. Published by The Company of Biologists under the terms of the Creative Commons Attribution License (https://creativecommons.org/licenses/by/4.0/).

discussion as possible mechanisms to explain their observations.

According to the reviewer's comment, we deleted the following sentences.

> If the instructive signal is directly transmitted from pR7 to R8, the signal transduction between rhabdomeres is probable, because their cell bodies are separated by the outer PR, R1. Rhabdomere is a specialized structure where Rhodospins and actin are densely accumulated, and Rh3 itself might act as an instructive signaling molecule. It should be noted that, in *dve*^{L186} mutant ommatidia, the instructive signal is almost completely blocked despite Rh3 expression in all R7s.

Another example pointed out by the reviewer was also changed to discriminate adult and pupal retina.

> **Our statement was toned down and the possibility of direct signaling from R7 to R8 is described as follows. Rhabdomeres of R7 and R8 are vertically aligned on the same axis and their cell bodies are separated by the outer PR, R1, in adult retina (Fig. 1A). However, cell bodies of R7 and R8 appear to contact directly during pupal development (Tomlinson, 1985). Thus, direct transmission of the instructive signal from pale-type R7 (pR7) to R8 is possible, whereas Dve expression is undetectable in these cells.**

Tomlinson, A. (1985) The cellular dynamics of pattern formation in the eye of *Drosophila*. Journal of Embryology and Experimental Morphology 89, 313-331.

NOTE: Figure provided for reviewer has been removed. It showed Figure 1 from Tomlinson, A. (1985) The cellular dynamics of pattern formation in the eye of *Drosophila*. *J Embryol Exp Morphol*. 89, 313-331. doi:10.1242/dev.89.1.313

We also changed the description in "Mechanisms of Rhodopsin coupling" as follows.
Our results suggest that the instructive signal from R7 activates Dve in adjacent R1 presumably through post-translational modification, and that the activated Dve (Dve*) disrupts the Hippo signaling pathway in adjacent R8.

We also added an introduction figure (Fig. 1A).

> **(A) Schematic representation of rhabdomere arrangement of two major ommatidial subtypes (pale and yellow). Rectangles and circles represent rhabdomeres and nuclei of photoreceptor cells (R1-R8 in white characters), respectively. Rhodopsin expression (Rh1,Rh3-Rh6) in rhabdomeres is shown in differently colored rectangles. Dve expression in nuclei is shown with magenta circles. The instructive Rh5-inducing signal from R7 to R8 is shown by a curved arrow. Horizontal sections of R7 and R8 layers show the position of rhabdomeres and cell bodies.**

A

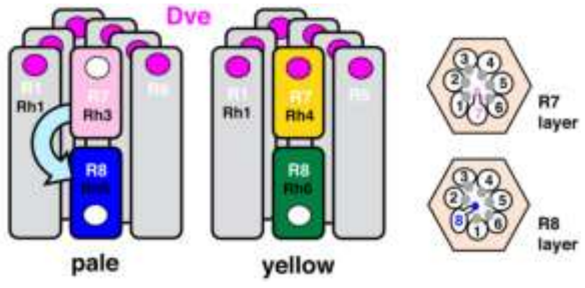

2. Alternative interpretations should be included: The authors only explore their single interpretation of their data. However, without additional experiments (which are otherwise not needed for this paper), they should provide alternative ideas about mechanisms driving these processes. These include:

a. Timing: The timing of the role of Dve could be important. How does the timing of Dve in outer PRs and R7s match up to the signaling event? Does Dve likely play a role in establishment and/or maintenance of signaling?

We added description about the timing of Dve action as follows.

© 2024. Published by The Company of Biologists under the terms of the Creative Commons Attribution License (https://creativecommons.org/licenses/by/4.0/).

If the Dve activity in R7 blocks the instructive signal, forced Dve expression in R7 should result in Rh6-expressing R8. As expected, forced Dve expression in all R7 of *ss* mutant eyes substantially blocked the instructive Rh5-inducing signal (Fig. S2). Incomplete inhibition of the instructive signal might be due to the timing of Dve expression, that the level of GAL4-mediated expression fluctuates among cells. Because GAL4 expression of *panR7-GAL4* is under the control of *rh3* and *rh4* promoters, the signal-sending activity of some R7 appears to be established before sufficient amount of Dve accumulates. Thus, *dve* loss-of-function and gain-of-function phenotypes are consistent with the notion that the Dve activity in R7 blocks the instructive signal.

b. dve mutant phenotypes could simply perturb coordination by derepression of signaling: The authors conclude that Dve in outer PRs plays a direct role in this process. The authors showed previously that Dve represses inner PR fate in the outers by repressing Rh3, Rh5, Rh6. In the outer PRs, Dve could simply be repressing signals and dve mutants yield derepression of these signals to perturb coordination of fates. To support the conclusion of the authors, they would have to ablate the cells and/or convert their fates and look at coordination, or determine what genes Dve regulates in outer PRs to control coordination. The authors should either provide these experiments OR provide alternative interpretations of their current data.

The instructive signal can be activated in the absence of R7 (newly added as Fig. 4A-D). If the outer cell fate is established by the Dve activity through repressing the signal (as a default state), R6 *dve* mutant can be explained as a relief of repression to induce Rh5. This is consistent with our observation.
In this scenario, Dve in R1 can activate "X" in Fig.4, and Dve should be inactivated in pale-type ommatidia to transmit the signal. However, in R1 *dve* mutant (inactivated Dve), the signal transmission is inhibited. So, we added the description for another possibility as follows.

As Dve represses Rh3, Rh5, and Rh6 in outer PR cells (Johnston et al., 2011), another possible mechanism is that Dve represses the instructive signal as a default state of outer PRs. In this case, the signal from R7 inhibits Dve in outer PRs and induces a relief of repression to induce Rh5. However, this is inconsistent with the result that R1 *dve* mutant completely blocks the signal. Thus, we propose a model that the presumptive active form of Dve (Dve*) in R1 is involved in the instructive Rh5-inducing signal (Fig. 4E).

3. The method to assess coordination is not clearly presented: In figure 3, the authors provide the data about coordination. However, they do not clearly describe how they did these experiments in terms of assessing cell fates. They authors should 1. provide a clear description of how they did the imaging and quantification and 2. Provide example images for all relevant phenotypes for all types of single cell clones.

We added Fig. S1 to describe how we get images as follows.

Fig. S1 Cell-specific *dve* mutant clones by the MARCM system
Cell-specific *dve* mutations are labeled with GFP expression (green) in the MARCM system (*GMR-flp/UAS-actGFP; FRTG13 GAL80/FRTG13 UAS-mCD8-GFP dve^{L186}; tub-GAL4/GMR-wIR*). Rhabdomeres are labeled with Phalloidin (red). (A) An ommatidium with R6-specific *dve* mutation. Dve (blue). The position of R1-R7 is numbered in A' and A''. Loss of Dve expression in R6 is indicated as a dotted circle in A''. (B-D) *dve* mutant ommatidia in the R7 layer (B) and the R8 layer (C). Rh4 (blue) and Rh5 (green) are shown. Ommatidia with typical Rh couplings are outlined with circles (pale and yellow, Rh3/Rh5 and Rh4/Rh6, respectively). Atypical types of ommatidia are outlined with squares (green and magenta, Rh3/Rh6 and Rh4/Rh5, respectively). Mutant R cells (R1, R6, and R7) and the dorso-ventral boundary of the equator (white line) are shown in (B) and the Rh coupling is shown in (C). (D) A magnified view of an ommatidium with R7-specific *dve* mutation in (B). (D') GFP expression in the R7 rhabdomere is indicated by an arrowhead.

Minor comments
1. The paper would benefit from an introduction figure to outline the ideas to the reader.

© 2024. Published by The Company of Biologists under the terms of the Creative Commons Attribution License (https://creativecommons.org/licenses/by/4.0/).

We added an introduction figure as Fig. 1A.

2. The retinas should be rotated 90° and shown with dorsal at the top.

We rotated the retina images in Fig. 2, Fig.4, and Fig. S2.

***** Reviewer 3 Advance Summary and Potential Significance to Field:
Sensory systems often rely on stochastic choices to express one sensory receptor gene out of many into each of the sensory neurons. The Drosophila fly eye serves as a particularly beneficial system to study the molecular events involved in stochastic choices because of its extreme simplicity (a binary stochastic choice), the deep knowledge of the system and the many tools available to study its molecular genetics. This paper takes advantage of this to propose a highly unexpected model that adds a surprising level of complexity to this apparently extremely simple system.
The choice in R7 cells to express Rh4 vs Rh3 is driven by the stochastic expression of spineless (ss) in Rh4-expressing cells. These cells repress Rh3 because Ss turns on another transcription factor, Dve in these cells and Dve represses Rh3. This is well established and a role of Dve restricted to repressing Rh3.
Where things become less clear is how R7 instructs the R8 cell below it to express Rh5 if R7 expresses Rh3 and Rh6 if R7 expresses Rh4. The latter is the default state since, in the total absence of R7 (sevenless), (almost) all R8 become Rh6.
The issue investigated here is why not only Rh4-R7 express Dve, but all outer photoreceptors R1-R6 also do express it.
photoreceptors R1-R6 also do express it.
Furthermore, the authors show that, in a null dve, ss double mutant, in which all R7 express Rh3 (and no dve), most R8 express Rh6: This means that Dve is involved in transmitting the signal from R7 to R8, but not by virtue of its expression in R7 since it is not expressed in the Rh3-R7.
The authors thus perform a clever experiment by benefiting from an old trick that allows one to mutate individually R7, R1 or R6, the last three cells born before the last mitosis.
The results are surprising:
- R1 dve mutants reproduce the whole eye null dve mutant: all R8 become Rh6, showing that the signal is conveyed by Dve in R1. How? This remains to be investigated
- More surprisingly, when R6 is mutant, now Rh5 is expressed in (many, not all) R8 below Rh4-R7 cells, suggesting that a signal mediated by R6 represses the Rh5 fate in ss ommatidia.
- Finally, when R7 is mutant for Dve in Rh4-R7, which now co-express Rh3 and Rh4 because Rh3 is no longer repressed, Rh5 is also coupled with Rh4
The conclusion is surprising as it shows that there is both a signal from Rh3-R7 to Rh5-R8 but there is also a signal to repress Rh5 in ommatidia that express ss. And both are mediated by Dve in R1, or in R6/R7! This is a striking result that should be published. Reviewer 3 Comments for the Author:
This is a striking result that should be published. I simply would like to suggest a few things:
- The paper has only three figures and a model. The authors must show several examples of R1, R6 and R7 mutant ommatidia instead than just the table of results: These experiments are often difficult to read and require a very good marking of the mutant cells. This would show that the data are very solid.

© 2024. Published by The Company of Biologists under the terms of the Creative Commons Attribution License (https://creativecommons.org/licenses/by/4.0/).

We added Fig. S1 to help better understanding of readers.

Fig. S1 Cell-specific *dve* mutant clones by the MARCM system
Cell-specific *dve* mutations are labeled with GFP expression (green) in the MARCM system (*GMR-flp/UAS-actGFP; FRTG13 GAL80/FRTG13 UAS-mCD8-GFP dve^{L186}; tub-GAL4/GMR-wIR*). Rhabdomeres are labeled with Phalloidin (red). (A) An ommatidium with R6-specific *dve* mutation. Dve (blue). The position of R1-R7 is numbered in A' and A''. Loss of Dve expression in R6 is indicated as a dotted circle in A''. (B-D) *dve* mutant ommatidia in the R7 layer (B) and the R8 layer (C). Rh4 (blue) and Rh5 (green) are shown. Ommatidia with typical Rh couplings are outlined with circles (pale and yellow, Rh3/Rh5 and Rh4/Rh6, respectively). Atypical types of ommatidia are outlined with squares (green and magenta, Rh3/Rh6 and Rh4/Rh5, respectively). Mutant R cells (R1, R6, and R7) and the dorso-ventral boundary of the equator (white line) are shown in (B) and the Rh coupling is shown in (C). (D) A magnified view of an ommatidium with R7-specific *dve* mutation in (B). (D') GFP expression in the R7 rhabdomere is indicated by an arrowhead.

- In the discussion, the authors mention "activation of Dve", which in one instance they correctly label Dve*. They should make sure that the readers understand that this is not transcriptional. In fact, Dve might be permissive here and simply allow the signal to be transmitted (or not) by R1 or R6.

We changed the description as follows.

The instructive signal from R7 activates Dve in adjacent R1 presumably through post-translational modification, and that the activated Dve (Dve*) disrupts the Hippo signaling pathway in adjacent R8.

- There were attempts to identify the signal from Rh3 to Rh5, which involves the BMP/Activin pathways, but this was difficult to interpret. The authors should revisit these data in view of their model in the discussion.

We deleted the following description.
Additional mechanism is also possible that BMP and Activin are secreted from pale R7 to the surface of adjacent R1, and that they are actively transferred to their receptors in R8. This transfer mechanism might be regulated by the Dve activity in R1.
We changed the description as follows.

Activin and BMP signaling are required upstream of the Hippo pathway to establish expression of Rh5 or Rh6 in R8 (Wells et al., 2017). If R7 secretes Activin and BMP ligands, these pathways might be independent of the Dve-mediated pathway because the instructive Rh5-inducing signal can be activated in the absence of R7 (Fig. 4).

- The paper is at time difficult to read for a non-specialist due to the confusion of nomenclature, R1, Rh3, Rh6, R6, R7...... The authors should make even more efforts to make these things clear

We added an introduction figure as Fig. 1A, and R cells are shown with white characters in Fig.1, Fig.3, and Fig.4 not to confuse Rh.

Fig.1 (A) Schematic representation of rhabdomere arrangement of two major ommatidial subtypes (pale and yellow). Rectangles and circles represent rhabdomeres and nuclei of photoreceptor cells (R1-R8 in white characters), respectively. Rhodopsin expression (Rh1, Rh3-Rh6) in rhabdomeres is shown in differently colored rectangles. Dve expression in nuclei is shown with magenta circles. The instructive Rh5-inducing signal from R7 to R8 is shown by a curved arrow. Horizontal sections of R7 and R8 layers show the position of rhabdomeres and cell bodies.

© 2024. Published by The Company of Biologists under the terms of the Creative Commons Attribution License (https://creativecommons.org/licenses/by/4.0/).

A

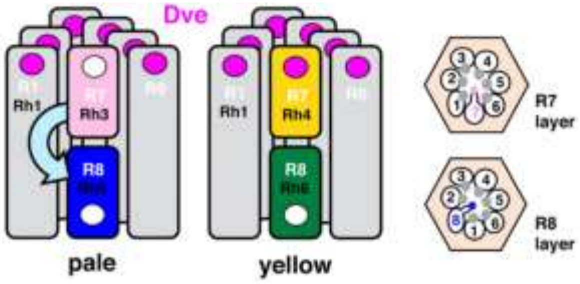

Second decision letter

MS ID#: DEVELOP/2023/202388

MS TITLE: Functional opsin patterning for Drosophila color vision is established through signaling pathways in adjacent object-detection neurons

AUTHORS: Manabu Kitamata, Yoshiaki Otake, Hideaki Kitagori, Xuanshuo Zhang, Yusuke Maki, Rika Boku, Masato Takeuchi, and Hideki Nakagoshi

ARTICLE TYPE: Research Report

I am delighted to tell you that your manuscript has been accepted for publication in Development, pending our standard ethics checks.

Reviewer 2

Advance summary and potential significance to field

The authors describe how motion-detecting photoreceptors are involved in the coordination of cell fates of color-detecting photoreceptors in the fly eye.

Comments for the author

The authors have addressed my concerns.

Reviewer 3

Advance summary and potential significance to field

Regulation of opsin expression

Comments for the author

The revised manuscript addresses in more detail the highly unexpected observation reported in the initial paper. The authors have responded well to the comments of the reviewers. They (the reviewers) would have liked to see more speculation as to how the system functions, but it is true that it appears quite complicated and difficult to fully understand Hopefully, the next paper will solve this.....
I strongly support publication of this paper

© 2024. Published by The Company of Biologists under the terms of the Creative Commons Attribution License (https://creativecommons.org/licenses/by/4.0/).
